# The Difference

**Published:** 1893-05

**Authors:** 


### THE DIFFERENCE.

When a simpleton wants to get well, he buys something "to take;" a philosopher gets something "to do;" and it is owing to the circumstance that the latter has been in a minority almost undistinguishable in all nations and ages, that doctors are princes instead of paupers; live like gentlemen, instead of cracking rocks for the turnpike.

